# Assessment of medical and dental students' knowledge, attitudes and practices related to fluoride exposure: a cross-sectional study from Jordan

**DOI:** 10.3389/froh.2026.1934956

**Published:** 2026-09-10

**Authors:** Hana Taha, Ruba AlHabahbeh, Mohammad Abdallah Alakhras, Rahmeh AlKharabsheh, Omar Wael Alhasan, Juman Wael Alhasan, Malek Awamleh, Shadi Al Khabazi, Suhib Awamleh, Fatima Taha, Linus Jönsson

**Affiliations:** 1 Department of Family and Community Medicine, School of Medicine, The University of Jordan, Amman, Jordan; 2 Department of Neurobiology, Care Sciences and Society, Karolinska Institutet, Stockholm, Sweden; 3 Department of Internal Medicine, King Hussein Cancer Center, Amman, Jordan; 4 Department of Dental Medicine, Universitetstandvården, Karolinska Institutet, Huddinge, Sweden; 5 College of Medicine, University of Sharjah, Sharjah, United Arab Emirates

**Keywords:** attitudes, dental students, fluoride exposure, Jordan, knowledge, medical students

## Abstract

**Background:**

Fluoride is protective against dental caries, but chronic overexposure causes dental and skeletal fluorosis and has been associated with neurological, endocrine, and renal effects. Jordan has endemic fluorosis, with dental fluorosis affecting 40%–70% of residents in several regions, yet no study has examined whether its future clinicians are prepared to recognize and manage fluoride toxicity. We assessed the knowledge, attitudes, and practices (KAP) of medical and dental students regarding fluoride exposure.

**Methods:**

In this cross-sectional study, 583 medical and dental students were recruited by convenience sampling at a single institution, the University of Jordan (June–December 2025), and completed a 27-item self-administered questionnaire adapted from a validated population-based instrument. It captured demographic and academic characteristics and three scored domains: Knowledge (range 0–14), Attitudes (5–25), and Practices (8–40). Domain scores were compared by program, gender, curriculum exposure, and year of study using t-tests and Welch's ANOVA with Games–Howell *post-hoc* tests, and independent predictors were identified with multivariable linear regression.

**Results:**

Mean Knowledge was low-to-moderate (5.38 ± 3.22; Cronbach's *α* = 0.806), with particularly poor awareness of non-dental complications, the WHO maximum permissible level (1.5 mg/L; correctly identified by 27.8%), and regulatory monitoring. Attitudes (16.92 ± 2.64) and Practices (22.15 ± 3.81) were broadly favorable but passive. Dental students and female participants scored higher across all three domains (*p* ≤ .025), and fluoride curriculum exposure showed direct association with Knowledge and Practices. Dental-program enrollment (*β* = 2.19) and detailed curriculum exposure (*β* = 4.84) were the strongest independent predictors of Knowledge which correlated most strongly with Practices (r = 0.410).

**Conclusion:**

Medical and dental students demonstrated limited knowledge of the systemic and regulatory dimensions of fluoride toxicity, and generally favorable attitudes did not translate into active protective behavior. Formal curriculum exposure showed the strongest association with preparedness, although the cross-sectional design does not permit causal inference. As the sample was drawn from a single institution and was overwhelmingly resident in the Central region, these findings should not be generalized to all Jordanian medical and dental students, particularly those in fluorosis-endemic areas. They nonetheless support targeted continuing education in fluorosis-endemic regions, and parallel investment in water safety.

## Introduction

Fluoride is a trace element that plays a significant role in dental health, particularly in the prevention of dental caries (1). It is found naturally in water, soil, and certain foods (e.g., seafood and tea), and is also added to oral care products like toothpaste and mouthwash (2). While fluoride's dental benefits are well recognized, increasing concerns have emerged over the potential harm overexposure can bring.

Excessive intake is associated with dental fluorosis, an irreversible enamel disorder ranging from discoloration to severe mottling and pitting of the teeth. Continued exposure can eventually lead to skeletal fluorosis, in which there is osteosclerosis, calcification of tendons and ligaments, and bone deformities (3). Neurotoxicity, endocrine disruption, and renal dysfunction have been associated with chronic fluoride exposure as well (2). A growing number of studies, including a major 2024 monograph by the National Toxicology Program (NTP), also suggest that exposure during pregnancy and early in life may be linked to reduced cognitive function in children (4–7).

These issues have raised public health concerns over fluoride and pushed some authorities to reexamine their policies surrounding it. Of these policies, community water fluoridation is widely adopted and represents a measure against dental caries. It is utilized by many countries to help reduce social inequalities in oral health access (8). However, the appropriateness of fluoridation depends on baseline groundwater levels, which vary considerably across regions (9). Groundwater is a major source of fluoride intake, and a high prevalence of dental fluorosis indicates excess exposure. Countries with either should be especially cautious. In areas endemic to fluorosis, water fluoridation should cease. Where levels are toxic, defluoridation may be needed to stay below the WHO limit of 1.5 mg/L (9, 10).

Jordan has a high prevalence of fluorosis, with high levels of groundwater contamination (9). The prevalence of dental fluorosis ranges from 40% to 70% in areas like Karak, Ruwaished and Kuraymah (11, 12). Despite this burden, no study has examined whether future healthcare providers in Jordan are adequately prepared to recognize and address fluoride toxicity in clinical practice. In this study, we assessed KAP levels among medical and dental students, whose responses reflect the current educational framework for this issue. We then compared these levels across program, year of study, and key demographic variables, and identified independent predictors of each domain. To our knowledge, this is the first study to examine this in Jordan.

## Methods

### Study design

This is a cross-sectional study conducted in the School of Medicine at the University of Jordan from June to December 2025. The data was collected using voluntary anonymous self-administered online questionnaire.

### Study setting

The University of Jordan has the biggest Medical and Dental program among all the universities in Jordan. It has around 5,000 medical students spread across 6 years of study and 2,000 dental students spread across 5 years. The academic year distribution is 9.5%, 12.3%, 15.5%, 13.7%, 10.1%, and 8.6% for first through sixth year medical students, respectively, and 3.4%, 6.3%, 7.9%, 7.2%, and 5.5% for first through fifth year dental students, respectively. This distribution was considered when evaluating the representativeness of the sample. Recruitment was restricted to this single institution because the year-of-study analysis required a cohort with a uniform, documented curriculum history, and because a single-site design was feasible within one institutional review board approval and the available resources. Universities in or serving the fluorosis-endemic governorates of northern and southern Jordan were therefore not sampled, and residents of those areas are represented only insofar as they had relocated to Amman to study. Eligible participants were students currently enrolled in the medical or dental program at the University of Jordan who were willing to participate; no additional exclusion criteria were applied.

### Measurement tool

The questionnaire was adapted from the previously validated Population-Based Knowledge Attitudes and Practices Questionnaire on Fluoride (PBKAP-QF), which originally demonstrated excellent internal consistency (Cronbach's alpha ranging from 0.884 to 0.910) (13). The questionnaire was adapted from the previously validated Population-Based Knowledge Attitudes and Practices Questionnaire on Fluoride (PBKAP-QF). The original instrument consisted of 19 items utilizing 3-point Likert scales. Deliberately minor modifications were implemented to optimize its application for this specific study population while preserving the tool's core psychometric integrity. First, minor linguistic adaptations were made to improve clarity. Second, the Knowledge domain was refined by adding one objective metric assessing awareness of the WHO's maximum allowed fluoride limits, and modifying an existing region-specific question by removing geographic references to broadly assess awareness of local public water supply monitoring. Third, the original 3-point Likert scales in the Attitudes and Practices domains were expanded to 5-point scales to increase response variance and sensitivity. Finally, a single compound item within the Practice domain regarding clinical consultation was separated into two distinct questions to accurately differentiate health-seeking behaviors for dental vs. skeletal complications. To ensure the integrity of the modified instrument, a two-step validation process was conducted. Content validity was established through expert review by senior faculty, and face validity was assessed via a pilot test conducted with a small sample of students to ensure the clarity of the modifications prior to broader distribution.

The questionnaire consisted of 27 items divided over four main sections. The first section captured demographic and academic characteristics, including age, gender, program of study, year of study, current place of residence, and prior exposure to fluoride-related content in the curriculum.

The second section assessed Knowledge across 8 items, covering self-rated knowledge about fluoride, awareness of natural and synthetic fluoride sources, dental and non-dental complications of chronic fluoride exposure, prevention measures to reduce fluoride exposure, the WHO maximum permissible fluoride level in drinking water (1.5 mg/L), and beliefs regarding regulatory monitoring of fluoride in public water supplies. Six items were rated on a 3-point scale (not aware/knowledgeable, somewhat aware/knowledgeable, very aware/knowledgeable), and two items were binary (yes/no or not sure).

The third section assessed Attitudes across 5 items measuring agreement that exceeding fluoride threshold levels constitutes a substantial public health concern, perceived severity of fluoride-related health complications, level of concern regarding personal exposure risk, belief that fluoride exposure can be reduced, and perceived effectiveness of water filters in mitigating fluoride toxicity. All items were rated on 5-point scales (two using a Strongly Disagree–Strongly Agree agreement format, and three using Not at all–Extremely severity/concern/effectiveness formats).

The fourth section assessed Practices across 8 items capturing the frequency of inquiring about water fluoride content, consuming filtered water, using fluoride-containing dental products, taking active measures to reduce fluoride intake, and seeking or sharing fluoride-related information, as well as the likelihood of seeking professional consultation if teeth discoloration or skeletal defects were observed in themselves or a family member. Six items used a 5-point frequency scale (Never–Always) and two items used a 5-point likelihood scale (Very unlikely–Very likely).

Domain scores were calculated by unweighted summation of item responses, with every item contributing equally to its domain total. In the Knowledge domain, the six 3-point items were scored 0 (not aware/knowledgeable), 1 (somewhat aware/knowledgeable) and 2 (very aware/knowledgeable), and the two binary items were scored 1 for the correct or affirmative response and 0 for an incorrect, negative or “not sure” response, giving a possible range of 0–14. In the Attitudes domain, all five items were scored 1–5 in the direction of the printed scale (1 = Strongly Disagree or Not at all; 5 = Strongly Agree or Extremely), giving a range of 5–25. In the Practices domain, all eight items were scored 1–5 (1 = Never or Very unlikely; 5 = Always or Very likely), giving a range of 8–40. All items were worded so that higher values denote greater knowledge, more favourable attitudes or more protective practices; no items were reverse-coded, and no weighting, standardization or imputation was applied. Participants with missing responses were not included, so all totals were computed on complete cases. Item wording, response options and item-level response frequencies for each domain are provided in Supplementary Tables SI–SIII.

### Sample size and data collection

Convenience sampling was used to recruit participants. The estimated minimum sample size was 384, calculated using Cochran's formula *N* = (Z^2^pq)/e^2^, where Z is the Z-score for a 95% confidence level (1.96), *p* is the expected prevalence (set at a conservative estimate of 50%, as no reliable prior prevalence estimate was available), and e is the margin of error (0.05). The survey link was distributed via class group chats and in-person QR codes. Because recruitment relied on broadly disseminated links rather than a closed sampling frame, an exact response rate could not be calculated. A total of 583 students were ultimately recruited to ensure broader representation across years and programs. This recruitment strategy has an acknowledged limitation: because sampling was non-probabilistic and confined to a single Amman-based institution, the resulting sample is geographically skewed toward the Central region (94.9%), with only 3.8% and 1.4% of participants resident in the North and South respectively. The sample therefore cannot be treated as representative of medical and dental students in Jordan as a whole, and in particular under-represents students from the fluorosis-endemic areas of the country. The implications of this for the interpretation and generalizability of our findings are examined in the Discussion.

### Data analysis

Descriptive statistics (means, standard deviations, frequencies, and percentages) were computed for all variables. Internal consistency of each domain (Knowledge, Attitudes, Practices) was assessed using Cronbach's alpha. Independent samples t-tests, with Levene's test for homogeneity of variances, were used to compare domain scores by program (medicine vs. dentistry) and gender, with Cohen's d reported as a measure of effect size. One-way Welch's ANOVA was used to compare domain scores across curriculum exposure groups, year of study (within each program), geographic region, and age group, with partial eta-squared (*η*^2^) reported as a measure of effect size, and Games-Howell *post-hoc* tests applied for pairwise comparisons. Pearson correlation coefficients were calculated among the three domain total scores. Multivariable linear regression models were constructed for each domain to identify independent predictors, using unstandardized coefficients (*β*), along with standardized coefficients and 95% confidence intervals. Prior to interpreting the regression models, assumptions were evaluated: multicollinearity was assessed using Variance Inflation Factor (VIF) and tolerance values (VIF ranged from 1.01 to 1.28 across all predictors, well within acceptable limits); homoscedasticity was assessed via plots of standardized residuals against fitted values, which showed no evidence of funneling or non-constant variance; normality of residuals was confirmed via Q-Q plots; and influential observations were screened using Cook's distance, the maximum value across all three models being 0.040, well below the conventional threshold of concern (Cook's D > 1). Statistical analyses were performed using jamovi (Version 2.7.31). Statistical significance was set at *p* < .05.

### Ethical considerations

This study was approved by the Institutional Review Board (IRB) of the Deanship of Scientific Research at the University of Jordan, Amman, Jordan (approval number 434/2025). Our study was conducted in accordance with the tenets of the Declaration of Helsinki. Participation was voluntary. All participants signed an electronic informed consent before they started filling out the questionnaire.

## Results

### Baseline characteristics

A total of 583 students completed the survey (Table 1). The majority were aged 18–21 years (*n* = 323; 55.4%), and most were female (*n* = 349; 59.9%). Medical students constituted 69.6% of the sample (*n* = 406), with representation across all six years of training; dental students comprised the remaining 30.4% (*n* = 177), spanning five years of study. Regarding prior fluoride-related curriculum exposure, 43.4% of participants reported none, 46.1% reported brief exposure, and 10.5% reported detailed coverage.

**Table 1:** Baseline demographic characteristics of participants (*N* = 583).

Variable	Category	*n*	%
Age	18–21 years	323	55.4
	22–25 years	257	44.1
	≥26 years	3	0.5
Gender	Male	234	40.1
	Female	349	59.9
Program	Medicine	406	69.6
	Dentistry	177	30.4
Year of Study	1st year	73	12.5
	2nd year	113	19.4
	3rd year	139	23.8
	4th year	136	23.3
	5th year	84	14.4
	6th year (Medicine only)	37	6.3
Region	North	22	3.8
	Central	553	94.9
	South	8	1.4
Fluoride Curriculum Exposure	None	253	43.4
	Yes, briefly	269	46.1
	Yes, in detail	61	10.5

### Knowledge

Item-level responses are presented in Supplementary Table SI. Self-reported awareness was highest for synthetic fluoride sources (35.2% “very aware”) but lowest for non-dental systemic complications and prevention measures (55.7% and 56.9% “not aware,” respectively). Only 27.8% of participants correctly identified the WHO maximum permissible fluoride level in drinking water (1.5 mg/L), and 63.6% did not believe, or were unsure, that authorities monitor fluoride levels in public water supplies. The mean total Knowledge score across all participants was 5.38 (SD = 3.22; possible range 0–14), indicating generally low-to-moderate knowledge of fluoride-related health issues. Reliability of the Knowledge scale was acceptable (Cronbach's *α* = 0.806). Dental students scored significantly higher than medical students [t(581) = −18.09, *p* < .001; Cohen's d = 1.63], and female participants scored higher than males [t(581) = −3.75, *p* < .001; Cohen's d = 0.32; Table 2]. Knowledge scores differed significantly across curriculum exposure groups in a graded pattern [F(2, 186) = 303.00, *p* < .001, partial *η*^2^ = 0.426], with all pairwise comparisons significant (Games-Howell, all *p* < .001; Table 2).

**Table 2:** Total KAP scores by program, gender, and curriculum exposure.

Variable	Category	Knowledge mean ± SD	Attitudes mean ± SD	Practices mean ± SD
Program	Medicine	4.11 ± 2.50	16.69 ± 2.67	21.69 ± 3.72
	Dentistry	8.31 ± 2.76	17.46 ± 2.49	23.19 ± 3.82
	*p*-value	<.001	.001	<.001
	Cohen's d	1.63	0.30	0.40
Gender	Male	4.78 ± 2.96	16.62 ± 2.56	21.56 ± 3.64
	Female	5.79 ± 3.33	17.12 ± 2.68	22.54 ± 3.88
	*p*-value	<.001	.025	.002
	Cohen's d	0.32	0.19	0.26
Curriculum Exposure	None	3.50 ± 2.28	16.51 ± 2.74	21.15 ± 3.50
	Yes, briefly	6.00 ± 2.69	17.13 ± 2.43	22.56 ± 3.70
	Yes, in detail	10.46 ± 1.93	17.75 ± 2.86	24.46 ± 4.23
	*p*-value	<.001	.002	<.001
	Partial *η*^2^	0.426	0.024	0.074

Values are Mean ± SD. *p*-values from independent samples t-tests (Program, Gender) or one-way Welch's ANOVA (Curriculum Exposure). b Games-Howell post-hoc: all pairwise comparisons for Knowledge and Practices significant (*p* ≤ .005); for Attitudes, significant differences between None vs. briefly (*p* = .018) and None vs. in detail (*p* = .008) only.

Within the medical program, Knowledge scores differed significantly by year of study [F(5, 160) = 7.24, *p* < .001], with 2nd-, 3rd-, and 4th-year students scoring significantly higher than 5th- and 6th-year students (all *p* ≤ .033). Among dental students, Knowledge scores increased progressively with year of study [F(4, 73.6) = 23.53, *p* < .001], with 4th- and 5th-year students scoring significantly higher than 1st- and 2nd-year students (all *p* ≤ .025; Table 3).

**Table 3:** Total KAP scores by year of study within each program.

Program	Year	*n*	Knowledge mean ± SD	Attitudes mean ± SD	Practices mean ± SD
Medicine	1st	54	3.63 ± 2.40	16.39 ± 2.98	21.54 ± 3.87
	2nd	75	4.83 ± 2.61	17.52 ± 2.81	21.75 ± 4.03
	3rd	98	4.46 ± 2.21	16.88 ± 2.60	21.45 ± 3.66
	4th	88	4.51 ± 2.75	16.56 ± 2.51	22.07 ± 3.95
	5th	53	2.98 ± 1.97	16.26 ± 2.07	21.74 ± 3.21
	6th	37	3.03 ± 2.33	15.84 ± 2.96	21.41 ± 3.27
	*p*-value		<.001	.029	.903
Dentistry	1st	19	7.11 ± 2.23	17.32 ± 2.31	21.89 ± 4.41
	2nd	38	6.11 ± 2.62	17.05 ± 2.13	22.03 ± 3.76
	3rd	41	7.73 ± 2.04	17.46 ± 2.05	23.71 ± 3.49
	4th	48	9.58 ± 2.46	17.33 ± 2.85	23.63 ± 3.84
	5th	31	10.55 ± 1.75	18.26 ± 2.86	24.06 ± 3.59
	*p*-value		<.001	.443	.091

Values are Mean ± SD. *p*-values from one-way Welch's ANOVA. Medicine: Knowledge *post-hoc* — 2nd, 3rd, and 4th years significantly higher than 5th and 6th years (*p* ≤ .033). Dentistry: Knowledge *post-hoc* — 4th and 5th years significantly higher than 1st and 2nd years (*p* ≤ .025).

### Attitudes

Item-level responses are shown in Supplementary Table SII. Most participants agreed or strongly agreed that exceeding the fluoride threshold is a substantial public health concern (67.4%) and that fluoride exposure is reducible (71.8%); perceived severity of health complications was rated “very” or “extremely” severe by only 31.4%.

In contrast, 47.5% of participants reported being “not at all” or only “slightly” concerned about fluoride exposure in their own daily lives. Water filters were rated “very” or “extremely” effective in reducing fluoride toxicity by only 27.4% of participants.

The mean total Attitudes score was 16.92 (SD = 2.64; possible range 5–25). Internal consistency was low (Cronbach's *α* = 0.387). Dental students scored significantly higher than medical students [t(581) = −3.29, *p* = .001; Cohen's d = 0.30], and female participants scored higher than males [t(581) = −2.25, *p* = .025; Cohen's d = 0.19; Table 2]. Curriculum exposure also had a significant effect [F(2, 164) = 6.41, *p* = .002, partial *η*^2^ = 0.024]: *post-hoc* tests showed significant differences between the “None” group and both exposed groups (*p* ≤ .018), but not between the two exposed groups (*p* = .256). No significant differences were found by year of study within either program.

### Practices

Item-level responses are presented in Supplementary Table SIII. Approximately half of participants reported never inquiring about fluoride content in drinking or cooking water (49.7%), seeking fluoride-related information from non-dental sources (50.1%), or discussing fluoride with friends or family (52.1%).

In contrast, 48.9% of participants reported always consuming filtered water daily, and 46.3% reported never or seldom using fluoride-containing dental products other than toothpaste.

Likelihood of seeking professional consultation was higher for skeletal defects (45.3% “very likely”) than for teeth discoloration (28.8% “very likely”).

The mean total Practices score was 22.15 (SD = 3.81; possible range 8–40). Internal consistency was low (Cronbach's *α* = 0.270). The lower values for attitudes and practices sections are expected in KAP inventories, as these domains measure a diverse portfolio of independent behaviors and perceptions rather than a single, highly correlated latent psychological construct. Dental students scored significantly higher than medical students [t(581) = −4.44, *p* < .001; Cohen's d = 0.40], and female participants scored higher than males [t(581) = −3.05, *p* = .002; Cohen's d = 0.26; Table 2]. Curriculum exposure was also associated with higher Practices scores [F(2, 163) = 20.75, *p* < .001, partial *η*^2^ = 0.074], with all pairwise comparisons significant (*p* ≤ .005). No significant differences were found by year of study within either program (Tables 2, 3).

### Correlations among KAP domains

Pearson correlation coefficients among the three KAP domain total scores are shown in Table 4. All three domains were positively and significantly inter-correlated (all *p* < .001). The strongest association was observed between Knowledge and Practices (r = 0.410), followed by Knowledge and Attitudes (r = 0.296) and Attitudes and Practices (r = 0.217).

**Table 4:** Pearson correlation matrix among KAP domain total scores (*N* = 583).

Variable	1. Knowledge	2. Attitudes	3. Practices
1. Knowledge	—		
2. Attitudes	0.296***	—	
3. Practices	0.410***	0.217***	—

*** *p* < .001.

### Multivariable linear regression

Three separate multivariable linear regression models were constructed to identify independent predictors of Knowledge, Attitudes, and Practices total scores (Table 5). The Knowledge model explained 49.1% of the variance [R^2^ = 0.491, F(4, 578) = 140, *p* < .001]. Enrollment in the dental program (*β* = 2.19, *p* < .001), detailed curriculum exposure (*β* = 4.84, *p* < .001), brief curriculum exposure (*β* = 1.69, *p* < .001), and female gender (*β* = 0.42, *p* = .032) were all independent positive predictors of Knowledge.

**Table 5:** Multivariable linear regression models for knowledge, attitudes, and practices.

Predictor	*β* (95% CI)	SE	Stand. β	*t*	*p*	R^2^
Knowledge Model						
Intercept	3.182 [2.82, 3.54]	0.183	—	17.42	<.001	0.491
Gender: Female vs. Male	0.422 [0.04, 0.81]	0.197	0.131	2.14	.032	
Program: Dentistry vs. Medicine	2.188 [1.66, 2.71]	0.266	0.679	8.22	<.001	
Curriculum: Brief vs. None	1.686 [1.24, 2.13]	0.225	0.523	7.51	<.001	
Curriculum: Detailed vs. None	4.836 [4.03, 5.65]	0.412	1.500	11.73	<.001	
Attitudes Model						
Intercept	16.266 [15.86, 16.67]	0.206	—	78.79	<.001	0.032
Gender: Female vs. Male	0.404 [−0.03, 0.84]	0.222	0.153	1.82	.070	
Program: Dentistry vs. Medicine	0.336 [−0.26, 0.93]	0.301	0.127	1.12	.265	
Curriculum: Brief vs. None	0.486 [−0.01, 0.99]	0.254	0.184	1.91	.056	
Curriculum: Detailed vs. None	0.852 [−0.06, 1.77]	0.466	0.323	1.83	.068	
Practices Model						
Intercept	20.588 [19.94, 21.23]	0.328	—	62.68	<.001	0.090
Gender: Female vs. Male	0.795 [0.18, 1.41]	0.313	0.209	2.54	.011	
Program: Dentistry vs. Medicine	0.106 [−0.72, 0.94]	0.422	0.028	0.25	.801	
Curriculum: Brief vs. None	1.328 [0.63, 2.03]	0.358	0.349	3.71	<.001	
Curriculum: Detailed vs. None	2.950 [1.64, 4.26]	0.667	0.774	4.42	<.001	
Age: 22–25 vs. 18–21	0.247 [−0.38, 0.87]	0.320	0.065	0.77	.441	
Age: ≥26 vs. 18–21	4.117 [−0.06, 8.30]	2.127	1.080	1.94	.053	

β, unstandardized regression coefficient. SE, standard error. R^2^ values reported for each model. Reference categories: Gender, Male; Program, Medicine; Curriculum, None; Age, 18–21 years.

The Attitudes model explained only 3.2% of the variance [R^2^ = 0.032, F(4, 578) = 4.79, *p* < .001], and no individual predictor reached the conventional significance threshold (all *p* ≥ .056).

The Practices model explained 9.0% of the variance [R^2^ = 0.090, F(6, 576) = 9.48, *p* < .001]. Female gender (*β* = 0.795, *p* = .011), brief curriculum exposure (*β* = 1.33, *p* < .001), and detailed curriculum exposure (*β* = 2.95, *p* < .001) were significant independent predictors. Study program and age group were not significant in this model.

## Discussion

This is the first assessment of fluoride-related knowledge, attitudes, and practices among medical and dental students in Jordan, a country where endemic fluorosis is well documented but where the training of future clinicians to recognize it has never been examined. While dental fluorosis has been documented in Jordan, our findings address the non-dental health implications of fluoride exposure and evaluate the preparedness of future healthcare providers to recognize and manage them.

Medical and dental students were targeted as the study population for their future role at the forefront of dealing with fluoride-related complications and counseling patients on them. Dental professionals have consistently been shown to possess greater fluoride-specific knowledge than physicians and other healthcare providers (14), a difference that follows naturally from the central place fluoride occupies in caries prevention and from the greater attention dental curricula give to fluorosis. However, awareness of non-dental complications was nonetheless low across the pooled sample.

Participants showed limited awareness of the non-dental complications of fluoride exposure, including skeletal fluorosis, neurotoxicity, endocrine and renal dysfunction. This held across both programs. The mechanisms behind these complications are increasingly well characterized. Fluoride accumulates in mineralized tissue and drives the osteosclerosis and ligamentous calcification of skeletal fluorosis (15), while experimental and epidemiological work implicates oxidative stress and disrupted neuronal signaling in its neurotoxic effects (16) and points to thyroid and renal involvement at sustained high intake (17, 18). That these pathways remain poorly recognized among students is concerning in a setting where the exposure is real and measurable. In Jordan, dental fluorosis affects 40% of residents of Karak, in the south, and has also been documented in Kuraymah and Ruwaished (11, 12). Two-thirds of adults in both regions had moderate to severe fluorosis (12). A visible, high burden of enamel disease is, in effect, a population-level marker of the same cumulative exposure that produces the systemic disease students struggled to identify (19). Early recognition of these manifestations depends on clinicians in both disciplines holding the full toxicity profile in mind, not only its cosmetic endpoint.

The low to moderate knowledge in our sample echoes the poor awareness of fluoride's health consequences reported in endemic Ethiopian communities (20), and the 69% of Saudi parents unaware of dental fluorosis or the harms of excessive intake (21). Comparable deficits have surfaced among health-professional trainees in other settings, which suggests the problem is one of health education rather than of any single population (22). The consistency of this pattern across very different populations matters most for endemic regions, where the cost of missed recognition is highest.

Awareness of the regulatory aspects of fluoride exposure was similarly limited. Most participants could not identify the WHO maximum permissible level in drinking water (1.5 mg/L), nor did they believe public water supplies are routinely monitored. Comparable gaps appear among practicing dentists in Kuwait, fewer than half of whom considered water fluoridation the most effective caries-prevention method (23). US physicians likewise hold inconsistent beliefs about fluoride delivery methods despite generally supporting water fluoridation (24). Read together, these results suggest that the public-health and regulatory framing of fluoride receives far less curricular attention than its clinical and cosmetic aspects, even within dentistry, where fluoride is otherwise emphasized (25).

The year-of-study pattern among medical students, in which second- through fourth-year students outperformed fifth- and sixth-year peers on Knowledge, is consistent with a specific curricular change at the University of Jordan. Content on fluoride was introduced approximately three years before data collection, and senior students did not encounter this material during their own preclinical years. This timing offers one plausible explanation for the pattern, but it cannot be established from our data. A cross-sectional design cannot separate a cohort effect of this kind from decay of knowledge over the course of training, and we did not collect process variables: course content, teaching hours, or instructional method, that would be needed to link the curricular change to the observed scores. The pattern is therefore best read as raising the possibility, rather than demonstrating, that curriculum reform may leave senior students and recent graduates uncovered (26).

However, dental students' Knowledge scores increased progressively and consistently with year of study. Fourth- and fifth-year students scored significantly higher than first and second year, suggesting that fluoride content is reinforced and built upon across the dental curriculum rather than delivered at a single point. This is in contrast to Muralidharan et al.'s (2018) study of dental students in South India where fluoride knowledge did not meaningfully improve from the preclinical to the internship year (27). The comparison is instructive. Exposure alone appears insufficient; it is the repeated reinforcement of a topic, rather than its one-time inclusion, that consolidates durable knowledge (28).

A striking dissociation emerged between how participants judged fluoride at the population level and how they judged it for themselves. Most accepted that exceeding the fluoride threshold is a genuine public-health concern, yet comparatively few expressed any personal worry about their own daily exposure. This gap between public and personal risk recognition is a recognized feature of health behavior. Risk-perception models account for it well: hazards that are invisible, delayed, and produce no immediate symptoms are readily discounted at the personal level even when their societal risk is acknowledged, a pattern consistent with optimistic bias (29). The limited awareness of systematic complications may compound this, making it difficult to perceive fluoride as a relevant individual health threat. This dissociation offers a coherent explanation for why higher Knowledge scores did not carry over into markedly better Practices, consistent with the moderate Knowledge–Practices correlation we observed (r = 0.410).

Practices scores reflected passive engagement with fluoride risk reduction. Approximately half the participants reported never inquiring about the fluoride content of their water, seeking information from non-dental sources, or discussing fluoride risks with others. This is consistent with dental students in South India, where positive fluoride attitudes did not predict proactive oral self-care behavior (27), This raises the possibility that the gap is characteristic of fluoride-related behavior in general rather than a peculiarity of our sample (30).

The one clear departure from this passivity was filtered-water consumption, reported as a daily habit by nearly half the sample (48.9%). This is more convincingly read as a byproduct of ordinary household water treatment in Jordan than as a deliberate anti-fluoride strategy. Domestic filtration and bottled water are widely used across the region for reasons of salinity, taste, and general potability rather than fluoride specifically (31, 32). This is supported by the absence of corresponding increases in fluoride-specific information-seeking or water-source modification among the same participants. Mitigation behaviors in fluorosis-endemic Ethiopian communities have similarly been attributed to household norms rather than health-specific motivation (20).

A notable behavioral discrepancy was the difference in healthcare-seeking behavior with dental vs. nondental manifestations of fluorosis. Participants were more likely to seek consultation for skeletal symptoms (45.3%) than for dental ones (28.8%), despite the fact that dental fluorosis is both the most prevalent and earliest-occurring manifestation of fluoride toxicity, appearing at substantially lower exposure thresholds than skeletal involvement (4). The likely driver is how the two presentations are weighed. Skeletal complaints carry immediate functional consequences, whereas enamel changes are read as cosmetic and quietly deprioritized. This is consistent with evidence that perceived symptom seriousness governs care-seeking, with symptoms judged non-serious prompting significant delay (33). Carried into clinical practice, this hierarchy is self-defeating, because it delays attention to precisely the sign that clinicians, dentists above all, are best positioned to catch first.

### Limitations

Although this study provides novel insights into the preparedness of future healthcare providers, several limitations must be acknowledged. The cross-sectional design precludes causal inferences regarding the impact of curriculum exposure; longitudinal tracking across academic years is required to confirm whether the observed knowledge deficits reflect true learning trajectories or simply cohort variations. Reliance on self-report introduces the familiar risk of social-desirability bias, which tends to inflate reported protective or socially approved behavior (34). If present here, it implies that real-world fluoride practices may be even more passive than our scores suggest, which only sharpens the case for behavioral intervention.

The low internal consistency of the Attitudes and Practices domains warrants comment. Whereas the Knowledge scale was robust (*α* = 0.806), the lower alphas in the two behavioral domains are consistent with greater item heterogeneity: attitudes and practices reflect several loosely related drivers (cultural norms, cost, convenience), so their items intercorrelate less tightly than knowledge items. Because alpha is sensitive to item heterogeneity and is not itself an index of dimensionality (35), these subscale totals are best interpreted as broad composite indicators rather than measures of a single latent trait. Total scores in these domains should therefore be read as broad composite indicators, and between-group differences carry more uncertainty than those in the Knowledge domain.

Finally, generalizability is constrained by the sample's geographic and institutional concentration. Because the vast majority of participants reside in the Central region and were recruited from a single university, the findings may not fully capture the awareness patterns of students from fluorosis-endemic areas in northern and southern Jordan. Additionally, while the unequal distribution of medical and dental students accurately reflects the institutional enrollment ratio rather than a sampling artifact, the pooled overall means are inherently weighted toward the medical cohort. Multi-center studies spanning several Jordanian universities and, ideally, sampling students drawn from endemic zones would give a more balanced and externally valid picture.

The direction and consequences of this geographic bias warrant separate consideration, because they bear directly on our principal conclusions rather than on generalizability alone. Our central finding — that knowledge of the non-dental consequences of fluoride exposure is low — rests on a sample in which 94.9% of participants reside in the Central region, an area not among those with documented endemic fluorosis. Plausible bias operates in two opposing directions. Students raised in Karak, Ruwaished or Kuraymah, where 40%–70% of residents show dental fluorosis, may have acquired experiential familiarity with fluoride and its manifestations that formal curricula do not supply; if so, the pooled Knowledge score understates the true cognitive proficiency of students from high-fluoride areas, and our conclusion is better read as specific to students without endemic-area exposure than as a national estimate. Conversely, endemic-area residence may normalize visible enamel change as an unremarkable local feature rather than a marker of toxic exposure, in which case awareness of systemic sequelae could be no higher, or lower, among such students. Our data cannot adjudicate between these possibilities: with 22 participants resident in the North and 8 in the South, the regional strata were too small to support a meaningful comparison of domain scores, so genuine geographical variation in fluoride-related knowledge, attitudes and practices (if it exists) would be obscured rather than detected by this design. The same constraint applies to the predictor models, in which region could not be entered as a covariate; the effects we attribute to program and curriculum exposure are therefore estimated within a largely Central-region population and may not hold uniformly across the country. For the same reason, our recommendations for targeted continuing education in endemic regions should be read as extrapolated from a non-endemic sample and treated as hypotheses for testing rather than as evidence-based prescriptions for those settings.

Future work should be designed specifically to resolve this. We propose a multi-centre study recruiting from institutions purposively selected to span the national fluoride-exposure gradient, including universities serving the endemic governorates of the north and south alongside Amman-based schools, with recruitment stratified by governorate of long-term residence rather than by institution, and strata sized *a priori* (using the standard deviations reported here) to detect a meaningful between-region difference in Knowledge score. Exposure should be classified objectively rather than by self-reported geography, by linking residential history to measured groundwater fluoride concentrations and, where feasible, clinical assessment of dental fluorosis using a standard index. Documenting fluoride curriculum content at each site would further allow curricular and environmental exposure, confounded in a single-site design, to be separated.

### Recommendations

Curriculum updating is a recognized approach to addressing knowledge gaps, although our design cannot confirm its effect in this setting, and older students and recent graduates may miss out on such updates and would benefit from supplemental educational materials, in the form of continued medical education (CME). Continuing education is an effective route for updating clinicians already in practice, particularly when it is interactive and delivered as repeated rather than one-off (36). Targeted, reinforced modules on fluoride toxicity could close the cohort gap that curriculum reform alone leaves open. Campaigns aimed at senior students, recent graduates, and the existing workforce are needed to raise awareness, correct entrenched misconceptions, and ensure that clinicians can recognize and manage both the dental and non-dental faces of fluorosis.

With water scarcity in Jordan and the poor socioeconomic status of some of the regions with endemic fluorosis, affected populations often don't have the option of seeking alternative sources of water and are left with a potentially contaminated water as their only source. Therefore, increasing awareness and preparedness of the workforce to deal with the complications arising from it is, albeit important, not enough and effort should be made to ensure the safety of the water they consume (37). Where groundwater fluoride exceeds the WHO limit, defluoridation and alternative-source provision are the only durable protections, and their feasibility in low-resource endemic settings deserves explicit policy attention (38).

The finding that 63.6% of participants were unaware of or uncertain about public fluoride monitoring in water supplies points to a transparency gap that health authorities should address by strengthening public reporting mechanisms and ensuring alignment with the WHO guideline.

Finally, a longitudinal tracking study is needed to verify the true relationship between curricular reform and changes in knowledge. Following successive cohorts through their training, with course content, teaching hours and instructional method documented as process variables, is the only design that can distinguish a genuine curricular effect from a cohort difference or from decay of knowledge across the years of study, which is a distinction our cross-sectional data cannot make. Future work should also assess whether educational interventions produce sustained KAP improvements, and should extend to other Jordanian universities, healthcare disciplines, and fluorosis-endemic regions, where the public health risks are the highest.

## Conclusion

This study found that medical and dental students at the University of Jordan demonstrate low-to-moderate knowledge of fluoride-related health issues, particularly regarding non-dental systemic complications, the WHO permissible threshold, and regulatory oversight of water supplies. Attitudes toward fluoride as a public health concern were broadly positive but did not translate into proactive protective behaviors, and Practices scores reflected largely passive engagement with fluoride risk reduction across both programs.

Dental program enrollment and fluoride-related curriculum exposure were the strongest independent predictors of Knowledge and Practices, indicating that formal education is strongly associated with health professional preparedness on this issue, although the direction of this relationship cannot be established from cross-sectional data. The year-of-study findings raise the possibility that senior students and recent graduates were not reached by recent curricular reforms and may benefit from targeted continuing education, although confirming this requires longitudinal data.

These results point to a broader pattern in which fluoride education for non-dental health professionals lags behind that of their dental counterparts, a disparity that warrants coordinated curricular, institutional, and public health responses, particularly in fluorosis-endemic regions where the clinical stakes are highest.

## Data Availability

The raw data supporting the conclusions of this article will be made available by the authors, without undue reservation.
