# Supplementary material for: Assessment of medical and dental students' knowledge, attitudes and practices related to fluoride exposure: a cross-sectional study from Jordan
Source: Front Oral Health. 2026 Sep 10;7:1934956. doi: 10.3389/froh.2026.1934956 (PMC13618689; doi:10.3389/froh.2026.1934956)
Supplement: Supplementary file 1 [file Table1.docx]

**Supplementary Tables**

**Supplementary Table I** *Item-Level Response Frequencies — Knowledge Domain (N = 583)*

| **Item** | **Not Aware / Knowledgeable n (%)** | **Somewhat Aware / Knowledgeable n (%)** | **Very Aware / Knowledgeable n (%)** |
| --- | --- | --- | --- |
| ***3-point scale items*** | | | |
| Self-rated knowledge about fluoride from a health-related perspective | 206 (35.3%) | 342 (58.7%) | 35 (6.0%) |
| Awareness of natural sources of fluoride (water, soil, food) | 225 (38.6%) | 298 (51.1%) | 60 (10.3%) |
| Awareness of synthetic sources of fluoride (toothpaste, mouthwash) | 67 (11.5%) | 311 (53.3%) | 205 (35.2%) |
| Awareness of dental fluorosis and its presentation | 179 (30.7%) | 198 (34.0%) | 206 (35.3%) |
| Awareness of non-dental complications of chronic fluoride exposure | 325 (55.7%) | 210 (36.0%) | 48 (8.2%) |
| Knowledge of prevention measures to reduce fluoride exposure | 332 (56.9%) | 205 (35.2%) | 46 (7.9%) |
| ***Binary items (Yes / No or Not Sure)*** | | | |
|  | **No / Not Sure n (%)** |  | **Yes n (%)** |
| Awareness of the WHO maximum permissible fluoride level in drinking water (1.5 mg/L) | 421 (72.2%) | — | 162 (27.8%) |
| Belief that authorities regularly monitor fluoride levels in public water supplies | 371 (63.6%) | — | 212 (36.4%) |

***Note.*** Binary items scored as Yes (1) or No/Not sure (0).

**Supplementary Table II** *Item-Level Response Frequencies — Attitudes Domain (N = 583)*

| **Item** | **1 n (%)** | **2 n (%)** | **3 n (%)** | **4 n (%)** | **5 n (%)** |
| --- | --- | --- | --- | --- | --- |
| ***Agreement scale (1 = Strongly Disagree → 5 = Strongly Agree)*** | | | | | |
| Exceeding the threshold level of fluoride exposure could pose a substantial public health concern | 24 (4.1%) | 20 (3.4%) | 146 (25.0%) | 322 (55.2%) | 71 (12.2%) |
| Fluoride exposure can be reduced | 14 (2.4%) | 23 (3.9%) | 127 (21.8%) | 329 (56.4%) | 90 (15.4%) |
| ***Severity / Concern / Effectiveness scale (1 = Not at all → 5 = Extremely)*** | | | | | |
| Perception of the severity of health complications caused by excessive fluoride intake | 28 (4.8%) | 82 (14.1%) | 290 (49.7%) | 155 (26.6%) | 28 (4.8%) |
| Concern about potential health risks associated with fluoride exposure in daily life | 125 (21.4%) | 152 (26.1%) | 195 (33.4%) | 84 (14.4%) | 27 (4.6%) |
| Belief that using water filters helps mitigate fluoride toxicity | 43 (7.4%) | 122 (20.9%) | 258 (44.3%) | 139 (23.8%) | 21 (3.6%) |

***Note.*** Items 1–2 used a 5-point agreement scale (1 = Strongly Disagree, 5 = Strongly Agree). Items 3–5 used a 5-point severity/concern/effectiveness scale (1 = Not at all, 5 = Extremely).

**Supplementary Table III** *Item-Level Response Frequencies — Practices Domain (N = 583)*

| **Item** | **Never n (%)** | **Seldom / Rarely n (%)** | **Sometimes / Occasionally n (%)** | **Often / Frequently n (%)** | **Always n (%)** |
| --- | --- | --- | --- | --- | --- |
| ***Frequency scale items*** | | | | | |
| Inquire about the fluoride content in drinking/cooking water | 290 (49.7%) | 120 (20.6%) | 123 (21.1%) | 39 (6.7%) | 11 (1.9%) |
| Consume filtered water daily | 50 (8.6%) | 52 (8.9%) | 94 (16.1%) | 102 (17.5%) | 285 (48.9%) |
| Use fluoride-containing dental products (other than toothpaste) | 126 (21.6%) | 144 (24.7%) | 181 (31.0%) | 88 (15.1%) | 44 (7.5%) |
| Make changes to drinking water sources or treatment to reduce fluoride intake | 313 (53.7%) | 100 (17.2%) | 92 (15.8%) | 45 (7.7%) | 33 (5.7%) |
| Seek information about fluoride from non-dental sources (e.g., health or environmental agencies) | 292 (50.1%) | 130 (22.3%) | 122 (20.9%) | 33 (5.7%) | 6 (1.0%) |
| Discuss or share information about fluoride in the environment with friends or family | 304 (52.1%) | 126 (21.6%) | 109 (18.7%) | 30 (5.1%) | 14 (2.4%) |
| ***Likelihood of seeking consultation (1 = Very unlikely → 5 = Very likely to consult a specialist)*** | | | | | |
| If teeth discoloration is observed in self or a family member | 36 (6.2%) | 143 (24.5%) | 128 (22.0%) | 108 (18.5%) | 168 (28.8%) |
| If skeletal defects (e.g., brittle bones, impaired joint mobility) are observed in self or a family member | 29 (5.0%) | 58 (9.9%) | 94 (16.1%) | 138 (23.7%) | 264 (45.3%) |

***Note.*** Items 1–6 used a 5-point frequency scale (1 = Never, 5 = Always). Items 7–8 used a 5-point likelihood-of-consultation scale (1 = Very unlikely, 5 = Very likely to consult a specialist).
