# Supplementary material for: Assessment of medical and dental students' knowledge, attitudes and practices related to fluoride exposure: a cross-sectional study from Jordan
Source: Front Oral Health. 2026 Sep 10;7:1934956. doi: 10.3389/froh.2026.1934956 (PMC13618689; doi:10.3389/froh.2026.1934956)
Supplement: Supplementary file 2 [file Table3.docx]

**Assessment of Medical and Dental Students’ Knowledge, Attitudes and Practices Related to Fluoride Exposure**

This study aims to examine the Knowledge, Attitudes and Practices of medical and dental students about fluoride intake. The data is anonymous and will be used for research purposes only. Your participation is voluntary, and you have the full autonomy to withdraw and stop your participation at any time.

I am aged 18 or above and I consent to participate in this research knowing that all the information that I will provide will be confidential and for research purposes only.

Supervisor: Dr. Hana Taha.

**Demographics**

**1. Age:**

18-21

22-25

26 and above

**3. Program of study:**

Medicine

Dentistry

**2. Gender:**

Male

Female

**4. Year of study:**

1.1st

2.2nd

3.3rd

4.4th

5.5th

6.6th

**5. Current place residence:**

1. Irbid
2. Ajloun
3. Jerash
4. Mafraq
5. Amman
6. Zarqa
7. Balqa
8. Madaba
9. Karak
10. Ma'an
11. Tafilah
12. Aqaba

**6. Have you studied fluoride-related topics in your curriculum?**

No

Yes, briefly

Yes, in detail

**Section 1: Knowledge**

**1. How knowledgeable are you about fluoride from a health related perspective?**

Not knowledgeable

Somewhat knowledgeable

Very knowledgeable

**2. Are you aware of any natural sources of fluoride (water, soil, and food)?**

Not aware

Somewhat aware

Very aware

**3. Are you aware of any synthetic sources of fluoride (e.g., toothpaste, mouthwash, or mousse)?**

Not aware

Somewhat aware

Very aware

**4. Are you aware of the maximum allowed level of fluoride in drinking water? (1.5 mg/L per WHO)**

Yes

No

Not sure

**5. Are you aware that excessive fluoride intake can lead to dental fluorosis, resulting in whitish-yellow or brown spots on teeth?**

Not aware

Somewhat aware

Very much aware

**6. Are you aware of non-dental complications of chronic fluoride exposure (e.g., skeletal and non-skeletal)?**

Not aware

Somewhat aware

Very much aware

**7. How knowledgeable are you about the prevention measures to reduce fluoride exposure?**

Not knowledgeable

Somewhat knowledgeable

Very knowledgeable

**8. To your knowledge, do authorities regularly monitor fluoride levels in public water supplies?**

Yes

No

Not sure

**Section 2: Attitudes**

**1. Exceeding the threshold level of fluoride exposure could pose a substantial public health concern.**

Strongly disagree

Disagree

Neutral

Agree

Strongly agree

**2. What is your perception of the severity of health complications caused by excessive fluoride intake?**

Extremely severe

Very severe

Moderately severe

Slightly severe

Not at all

**3. How concerned are you about potential health risks associated with fluoride exposure in your daily life?**

Extremely concerned

Very concerned

Moderately concerned

Slightly concerned

Not at all

**4. Fluoride exposure can be reduced.**

Strongly disagree

Disagree

Neutral

Agree

Strongly agree

**5. Do you believe that using water filters helps mitigate fluoride toxicity?**

Not at all

Slightly effective

Moderately effective

Very effective

Extremely effective

**Section 3: Practice**

**1. How often do you inquire about the fluoride content in the water you use for drinking and cooking?**

Never

Seldom

Sometimes

Often

Always

**2. How frequently do you consume filtered water daily?**

Never

Seldom

Sometimes

Often

Always

**3. Other than toothpaste, how frequently do you use fluoride-containing dental products (gel, mouthwash, etc)?**

Never

Seldom

Sometimes

Often

Always

**4. Have you ever made changes to your drinking water sources or implemented water treatment methods to reduce fluoride intake due to health concerns?**

Never

Rarely (only once or twice)

Occasionally (a few times)

Frequently (made repeated changes or treatments)

Always (consistently use water treatment or alternative sources)

**5. How often have you sought information or education about fluoride from non-dental sources, such as environmental or health agencies?**

Never

Seldom

Sometimes

Often

Always

**6. How often do you discuss or share information about the natural occurrence of fluoride in the environment with friends or family?**

Never

Seldom

Sometimes

Often

Always

**7. If you observe any teeth discoloration in yourself or a family member, how likely are you to seek consultation or professional advice?**

Very unlikely to seek help

Unlikely, but might consider it if the condition worsens

Neutral/ not sure

Likely to consult a general healthcare provider

Very likely to consult a dental specialist

**8. If you observe any skeletal defects (e.g., brittle bones and impaired joint mobility) in yourself or a family member, how likely are you to seek consultation or professional advice?**

Very unlikely to seek help

Unlikely, but might consider it if the condition worsens

Neutral/ not sure

Likely to consult a general healthcare provider

Very likely to consult a medical specialist
